# Experiences and attitudes of UK early career psychiatrists towards electroconvulsive therapy

**DOI:** 10.1192/j.eurpsy.2024.1658

**Published:** 2024-08-27

**Authors:** L. Alexander, G. Bhatia, M. Pinto da Costa

**Affiliations:** ^1^South London and the Maudsley NHS Foundation Trust; ^2^Institute of Psychiatry, Psychology and Neuroscience, King’s College London, London, United Kingdom; ^3^Institute of Biomedical Sciences Abel Salazar, University of Porto, Porto, Portugal

## Abstract

**Introduction:**

Electroconvulsive therapy (ECT) is effective in treating severe major depressive disorder, manic episodes, and catatonia. Despite this, it is a controversial treatment amongst patients, carers, and even some psychiatrists in the UK.

**Objectives:**

To determine the experiences and perceptions of UK psychiatric trainees and early-career psychiatrists regarding the use of ECT in clinical practice.

**Methods:**

An anonymous survey was distributed online to UK psychiatric trainees and early-career psychiatrists across the country. The questionnaire consisted of 36 multiple-choice and Likert scale questions.

**Results:**

So far, 44 trainees and early-career psychiatrists have responded. The vast majority had witnessed ECT administration during training and had administered ECT under supervision. Most respondents agreed or strongly agreed that ECT was a safe and effective treatment, and most respondents disagreed or strongly disagreed that ECT is cruel or outdated. There were more varied views regarding perceptions of side effects and contraindications: a minority of respondents were unsure about whether ECT had long-term side effects, and whilst most respondents disagreed or strongly disagreed that ECT has many risks and contraindications, just under half were unsure or agreed.

**Conclusions:**

Most UK psychiatric trainees and early-career psychiatrists have experience of ECT during training and believe ECT is a safe and effective treatment. Respondents had a mixed view regarding the side-effect profile and risks/contraindications of ECT, which may be an important area for further education and training.

**Disclosure of Interest:**

None Declared

